# Quantum Mechanical Investigation of the Oxidative Cleavage of the C–C Backbone Bonds in Polyethylene Model Molecules

**DOI:** 10.3390/polym13162730

**Published:** 2021-08-15

**Authors:** Qixuan Jiang, Zhongyu Li, Ziheng Cui, Ren Wei, Kaili Nie, Haijun Xu, Luo Liu

**Affiliations:** 1 Beijing Bioprocess Key Laboratory, Beijing University of Chemical Technology, Beijing 100029, China; 18021336295@163.com (Q.J.); 2020201142@buct.edu.cn (Z.L.); cuileft@gmail.com (Z.C.); niekl@mail.buct.edu.cn (K.N.); 2 Junior Research Group Plastic Biodegradation, Department of Biotechnology and Enzyme Catalysis, Institute of Biochemistry, University of Greifswald, Felix-Hausdorff-Straße 4, D-17487 Greifswald, Germany; ren.wei@uni-greifswald.de

**Keywords:** plastic degradation, oxidation, free radicals, C–C bond cleavage

## Abstract

Recalcitrant plastic waste has caused serious global ecological problems. There is an urgent need to develop environmentally friendly and efficient methods for degrading the highly stable carbon skeleton structure of plastics. To that end, we used a quantum mechanical calculation to thoroughly investigate the oxidative scission of the carbon-carbon (C–C) backbone in polyethylene (PE). Here, we studied the reaction path of C–C bond oxidation via hydroxyl radical in PE. The flexible force constants and fuzzy bond orders of the C–C bonds were calculated in the presence of one or more carbocations in the same PE carbon chain. By comparison, the strength of the C–C bond decreased when carbocation density increased. However, the higher the density of carbocations, the higher the total energy of the molecule and the more difficult it was to be generated. The results revealed that PE oxidized to alcohol and other products, such as carboxylic acid, aldehyde and ketone, etc. Moreover, the presence of carbocations was seen to promote the cleavage of C–C backbones in the absence of oxygen.

## 1. Introduction

Plastics are synthetic polymers that aid in the construction of the modern world [1]. Now, plastics symbolize a disposable lifestyle for the majority of the general public and are associated with low-quality and low-value products [2]. In 2018, the global production of plastics nearly reached 360 million tons [3]. Despite the benefits, the use of plastics is causing significant environmental problems [4,5]. Plastic waste pollutes the natural environment, with microplastics and nanoplastics omnipresent in many ecosystems [6]. The ubiquitous contamination of the environment by microplastics and nanoplastics, along with the potential risks to ecosystems and ultimately to human health, has recently received an abundance of scientific and public attention [7].

The visible evidence of plastic pollution, as well as the currently unknown impacts of these materials, have prompted a rethinking of technical solutions. Plastic degradation in the environment can be divided into two categories: abiotic degradation (thermal degradation, photodegradation and radiation degradation) and biodegradation [8]. Future end-of-life options have been envisioned with plastic litters possessing value and useful properties. Here, biodegradation are considered to play a key role [9]. The majority of conventional petrochemical-derived plastics are non-hydrolysable vinyl polymers with saturated carbon-carbon (C–C) backbones, such as polyethylene (PE), polystyrene (PS) and polyvinyl chloride (PVC) [1]. *Aspergillus flavus* VRKPT2 have been found to contribute to the degradation of HDPE waste, resulting in a weight loss of 8.51 ± 0.1% after 30 days of incubation [10]. Li et al. found that the marine bacterium *Microbulbifer hydrolyticus* IRE-31, detected from lignin-rich wastewater, effectively degraded LDPE [11]. Among the reported microorganisms capable of degrading PE are the following fungi: *Aspergillus niger*, *Aspergillus flavus*, *Aspergillus oryzae*, *Chaetomium globusum*, *Penicillium funiculosum* and *Pullularia pullulan*. As for bacteria, the list includes *Pseudomonas aeruginosa*, *Bacillus cereus*, *Coryneformes bacterium*, *Bacillus* sp., *Mycobacterium*, *Nocardia*, *Corynebacterium*, *Candida*, *Pseudomonas* and *Actinomycetales* (*Streptomycetaceae*) [12]. These findings suggest that certain microbial strains can be used to degrade plastic waste. However, only a handful of microbial enzymes that degrade PE and PS have been so far reported on. Santo et al. isolated the actinomycete *Rhodococcus ruber* to degrade PE and demonstrated that laccase played a crucial role in the oxidation and degradation of PE [13]. Aside from laccases, several enzymes have been identified as being involved in the biodegradation of PE and PS, including the alkane hydroxylase AlkB [14,15] and a hydroquinone peroxidase [16]. 

To determine the biocatalytic degradation of the inert C–C backbones, Xu et al. investigated the catalytic mechanism of P450 monooxygenases by quantum mechanical calculation, suggesting that the oxygenase-induced free radical transition caused the C–C bond cleavage in aliphatic compounds [17]. The majority of non-hydrolysable polymer degradation follow the same basic oxidation mechanism mediated by radicals [18]. This catalytic degradation process is assumed to be common for most hydrocarbons, leading to hydroperoxide propagation, hydrogen abstractions, rearrangements of macroradicals and, ultimately, polymer degradation through the introduction of functional groups and C–C backbone cleavage [19,20]. 

While much progress has been made with regard to understanding the enzymatic degradation of polyesters, the mechanisms and limiting factors for the biocatalytic degradation of plastics with C–C backbones remain unknown [21]. Monitoring of active intermediates such as free radicals and carbocations is required to better understand the mechanisms involved in C–C backbone scission by oxidation at the molecular level, which is difficult to perform using standard experimental methods. The current study investigated the intermolecular reactivity of PE with hydroxyl radicals and the effects of carbocations on the carbon skeleton of PE using quantum mechanical calculations based on the chain-flexibility hypothesis. This study aimed to provide fundamental insights into the reaction mechanisms for the enzymatic PE degradation by oxidation, which can facilitate the further development of biodegradation methods for plastic waste.

## 2. Calculation Methods

### 2.1. Computer Programs for QM Calculations 

When studying the reaction of PE with hydroxyl radical and the influence of carbocations on carbon chain strength, the Gaussian 09 software package (Gaussian, Inc. Wallingford, CT, USA) [22] was used to perform a series of calculations on the molecules including geometric optimization, transition state search, intrinsic reaction coordinate (IRC) [23,24] analysis and flexible scanning, etc. In the computational process, geometric optimization and frequency analysis were carried out for each molecular structure at the same calculation level, based on the density functional theory at the B3LYP/6−31+G(d,p) theoretical level, to ensure that there was no imaginary frequency when the molecule was at a stable minimum in the potential energy landscape and only had one imaginary frequency for transition state. The calculation of molecular spin density, fuzzy bond level, potential energy surface intersection points of different spin multiplicity of the same molecular structure and flexible force constants were calculated with the help of the program Multiwfn [25] (i.e., quantum chemical wave function analysis program), sobMECP2 (http://sobereva.com/286) 10 August 2021 and compliance (http://www.oc.tu-bs.de/Grunenberg/index.html) 10 August 2021 [26,27].

The wave function in quantum mechanics describes the quantum state of a system of one or more particles. It contains a wealth of information about the system when considered in isolation. By analyzing the wave function and its derivative information (such as electron density), we can thoroughly understand the inherent characteristics of the system, such as the strength and nature of the bonds between atoms, the behavior and distribution of electrons in the system, etc. Simultaneously, we can also predict how the current system interacts with other systems, such as predicting reaction sites.

### 2.2. Bond Strength Descriptors 

Bond dissociation energy (BDE)—i.e., the enthalpy change of bond fracture—is widely used to describe a bond strength. However, BDE as an intrinsic strength of a particular bond depends on the stable molecule and the stability (e.g., electronic ground state, minimum conformation, etc.) of the fragments [28].

The bond order is another quantitative descriptor that has been used to understand molecular electronic structure and to predict reactivity, aromaticity and stability [29]. The physical essence of fuzzy bond order is to measure the number of shared electrons between atoms, which directly reflects the degree of delocalization of electrons between two atomic spaces [30]. The fuzzy bond order exhibits very little basis set sensitivity and does not deteriorate when using diffusion basis functions [31]. 

The flexible force constant is equivalent to the force constant in the direction of bond expansion and contraction, which is considered to be a more rigorous method for examining the strength of chemical bonds than bond energy, and is often used as a direct measure of the physical quantity of bond strength [26,32].

## 3. Results and Discussion 

### 3.1. Oxidation of PE by Free Oxygen Radicals

**Scheme 1:**
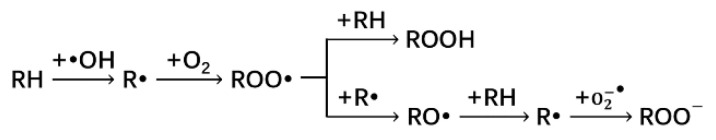
A summary of reaction pathways leading to oxidation of alkane.

In Scheme 1, we summarize the likely reaction pathways that leads to polyethylene oxidation.

#### 3.1.1. Reaction of Alkane with Hydroxyl Radical

PE is a large macromolecule. Therefore, quantum chemical calculation for reactions with PE main chain exceed the computation capacity of most supercomputers. Instead, in this study, C_6_ alkane molecules are selected to resemble a fragment in a real PE molecule.

**Figure 1:**
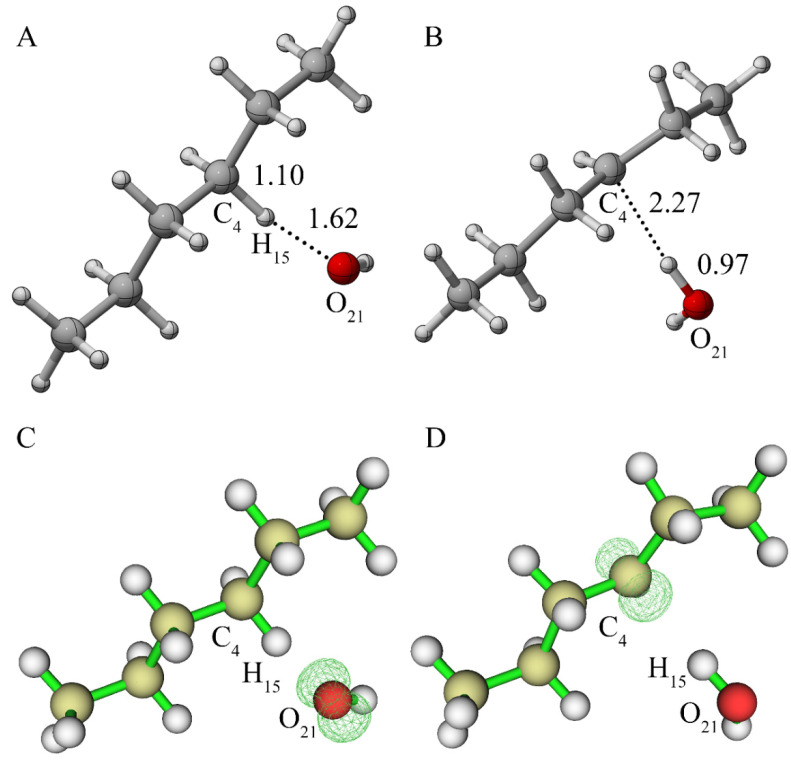
(**A**) Structure of alkane and •OH radical system before geometry optimization; (**B**) structure of alkane and •OH radical system after geometry optimization; (**C**) spin density of molecular structure of alkane and •OH radical system before geometry optimization; (**D**) spin density of molecular structure of alkane and •OH radical system after geometry optimization (the value of the iso-surface of (**C**,**D**) is 0.01).

Firstly, we studied the reactivity of alkanes with •OH radicals. In a vacuum, the energetically optimized alkane and •OH radical structures were used to generate a basic molecular system (Figure 1A), which was then geometrically optimized for the lowest free energy in a vacuum (Figure 1B). The distances between C_4_ and H_15_, and O_21_ and H_15_, were changed from 1.10 Å to 2.27 Å, 1.62 Å to 0.97 Å, respectively, after optimization, in which the radical was transferred to C_4_ in the alkane. This resulted in a free molecule of water. Afterwards, we performed the spin density analysis of the molecular system. As shown in Figure 1D, the single electrons of the entire system were mainly distributed on the carbon backbone. 

Since a hydrogen atom at the C_4_ position in the optimized molecular structure was directly transferred to the OH radical, the reaction of the alkane and the OH radical was likely to be spontaneous. To investigate this reaction more clearly, the distance between O_15_ and H_15_ was taken as the abscissa to perform a flexible scan. As shown in Figure S1, the energy of the entire system decreased rapidly due to the distance between O_15_ and H_15_ from 0.50 up to approximately 1.00. It then slightly decreased as the distance increased further to 2.00 Å. This spontaneous abstraction of hydrogen from the carbon backbone of alkane was consistent with the findings reported by Mayer et al., who indicated that H-abstraction reactions of propylene carbonate are thermodynamically favorable (i.e., exothermic and spontaneous in nature) [33]. A similar calculation was found elsewhere in the literature [34], wherein an abstraction of the hydrogen atom was seen to initiate a series of subsequent cleavage reactions of PE. 

**Figure 2:**
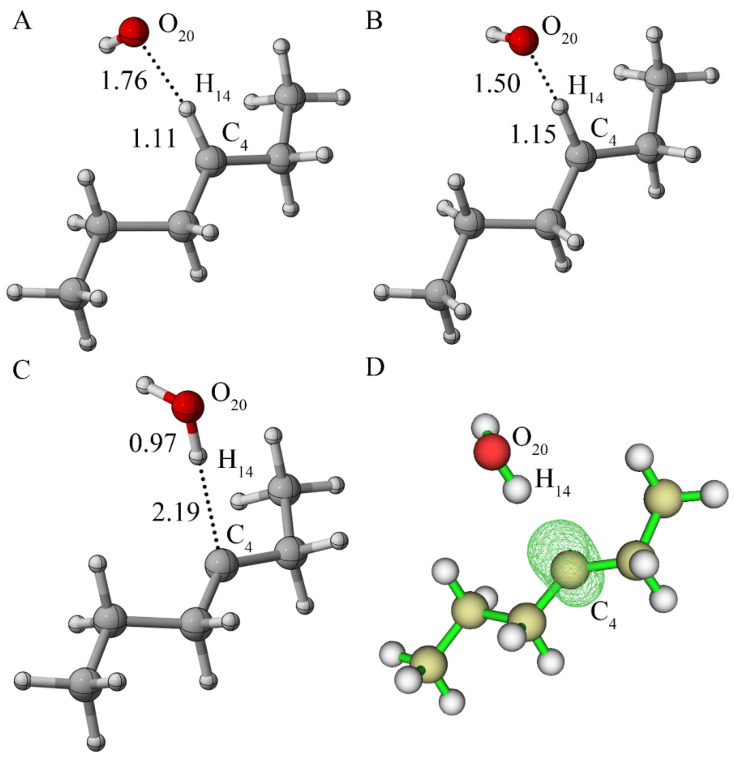
(**A**) Reactants containing an alkane radical and a OH radical; (**B**) transition state; (**C**) products; (**D**) spin density of the structure as shown in (**C**) (the value of the iso-surface is 0.01).

We then explored whether another hydrogen atom on the same carbon atom (C_4_) in the alkane backbone could further be abstracted by a second OH radical. The reactants, transition state, products and their spin density of the above reaction are shown in Figure 2. Figure S2 shows the energy curve obtained via IRC analysis. Figure 2D indicates that the α electrons are basically concentrated at the C_4_ position where the H_14_ atom was abstracted. Similar to the above results, the hydroxyl radical captured the hydrogen atom, resulting in the formation of a water molecule. After calculating the energy difference between the transition state and the reactants, the activation energy was determined to be almost zero, indicating that the two hydrogen atoms on the same carbon atom can be easily abstracted by hydroxyl radicals one after another.

The influence of successive H-abstraction on C–C bond was also studied; the optimized structures are shown in Figure S3. The fuzzy bond order of C–C bonds in different alkane radicals was calculated in order to study the influence of H-abstraction (Table 1). The bond order of each investigated C–C bond did not significantly change, which clearly indicates that the formation of alkane radicals does not lead to a direct cleavage of the carbon backbone. However, the formed carbon radical in PE can react with oxygen, thereby facilitating the oxidation of PE [34]. 

The above results indicate that hydrogen atoms can be easily abstracted by OH radicals, but the formation of alkanes radicals do not facilitate the direct cleavage of carbon backbones. Therefore, other oxidizing substances are required for the chain breakdown. Oxygen was the next considered option because of its widespread presence in the natural environment and its industrial application value. Moreover, superoxide anion radical was considered due to its strong oxidizing property.

#### 3.1.2. Reaction of Alkane Free Radical with Oxygen

Shi et al. found that the reaction of long-chain alkanes with OH radicals are mainly through H-abstraction to generate alkyl radicals. These alkyl radicals are then subjected to a series of free radical reactions to generate a variety of oxygen-containing organic compounds [35]. Similarly, it was reported by Bertin et al. that in the presence of oxygen, alkyl radicals formed after fragmentation of alkoxyl radicals in polypropylene, which were scavenged by the oxygen to afford peroxyl radicals [36]. Therefore, it was further investigated whether the alkane radical can react with oxygen. The following reactions was thus formed:

R· + O_2_→ROO·

Because molecular oxygen is a biradical in triplet state, it is not easy to calculate reactions involving this species. If the spins of reactants and products in a reaction are different then there must be intersections between the potential energy surfaces of reactants and products. The transition state energy of this reaction depends on the smaller value of the transition state energy on the two potential energy surfaces. 

**Figure 3:**
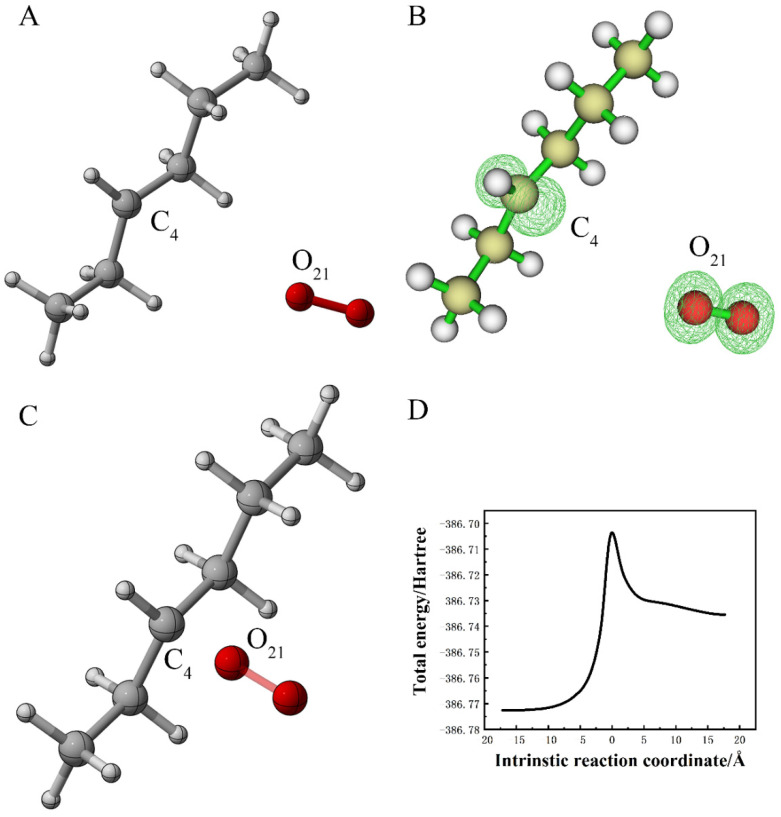
(**A**) Reactant of alkane radical with oxygen in the quadruple state; (**B**) spin density of the structure as shown in (**A**) (the value of the iso-surface is 0.01); (**C**) transition state; (**D**) IRC analysis potential energy curve.

Firstly, the reaction of alkane radicals with oxygen in the quadruple state was studied, as shown in Figure 3. Figure 3B illustrates that the unpaired electrons were all α electrons, which was mainly distributed on the C_4_ atom. There were three unpaired electrons found with the same spin direction, which indicated that the system was in the quadruple state. The activation energy, as indicated in Figure 3D, was 0.07 Ha (equal to 44.93 kcal/mol), which was too high for the reaction to occur.

**Figure 4:**
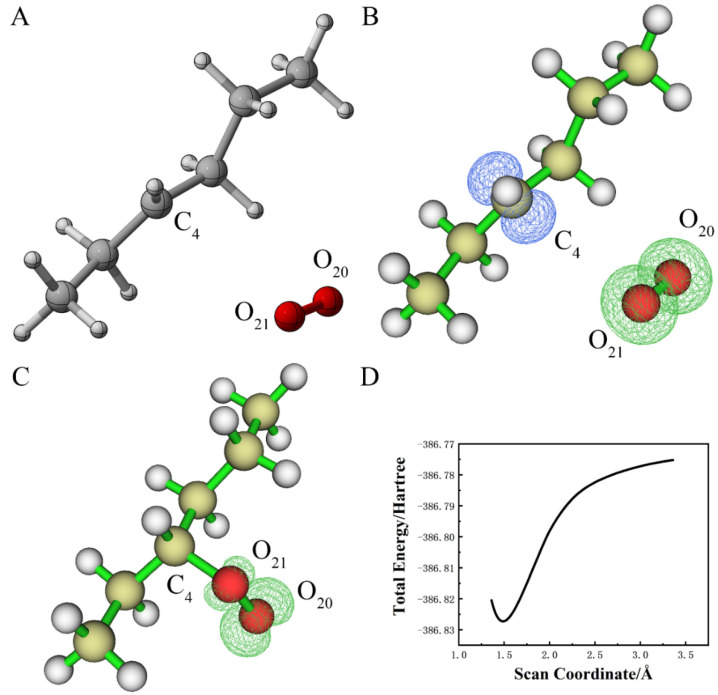
(**A**) The structure closest to the reactant of alkane radical with oxygen in the double state (when distance between O_21_ and C_4_ is the farthest during the relaxed scan); (**B**) the spin density of the structure in (**A**); (**C**) the spin density of the structure after the geometry optimization of the lowest energy point structure during the relaxed scan (this is a reflexive reaction with no transition state); (**D**) the result of the relaxed scan illustrates the change of the total energy along with the distance between O_21_ and C_4_.

Secondly, the reaction of alkane radicals with oxygen in the double state was studied, as shown in Figure 4. Moreover, a flexible scanning was performed along the direction of the line connecting O_21_ and C_4_. Figure 4B shows that the unpaired electrons were mainly distributed on the oxygen molecule and C_4_. The spin direction of the unpaired α electron on the oxygen molecule was opposite the unpaired β electron on C_4_, indicating that the structure was in a doublet state. The curve in the Figure 4D indicates that as the distance between O_21_ and C_4_ atom increased, the energy of the system went down first, then up quickly. This indicates that in the doublet state, the reaction between oxygen molecule and alkane radical is spontaneous. The spin density of the reaction state with the lowest energy is shown in Figure 4C. Here, the unpaired electrons were mainly distributed on two oxygen atoms. Further, the density of unpaired electrons O_20_ was higher than that of O_21_. The unpaired electrons were all α electrons and their spin directions were the same, whereas the unpaired β electrons in Figure 4B disappeared. From the above results, it is clear that the unpaired α electrons and β electrons were combined to form a new C-O bond, which explains why the reaction of the oxygen molecule and the alkane radical was spontaneous in the double state. 

**Figure 5:**
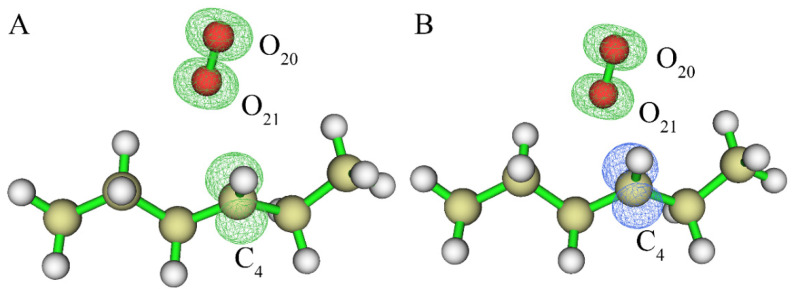
(**A**) The spin density of the minimum energy crossing point in the quadruple state; (**B**) the spin density of the minimum energy crossing point in the double state (the value of the iso-surface is 0.01).

The program sobMECP2 was used to calculate the minimum energy intersection point of the system on two potential energy surfaces with a different spin multiplicity. The energy of the intersection in the quadruple state in Figure 5A was −386.77 Ha, in the double state; in Figure 5B it was −386.77 Ha. Moreover, the energy of the structure in Figure 3A was found to be −386.77 Ha. All told, this revealed that the energy of the reactant and the energy of the intersection was close, and the barrier between the two energies was almost zero. In 2019, Ward et al. reported that polystyrene could be completely oxidized to carbon dioxide and partially oxidized in order to dissolve organic carbon under the participation of light and oxygen [37]. Jiao et al. reported a highly selective conversion of various waste plastics into C_2_ fuels under simulated natural environments via a designed photoinduced sequential C−C cleavage and coupling pathway, where polyethylene, polypropylene and polyvinyl chloride could be photocatalyzed into CH_3_COOH without using sacrificial agents [38]. Under the condition of natural light, energy is absorbed by the reactant system composed of alkane and oxygen molecule causing electronic transition and transfers to excited state. In this way, the structure of the reactant changes to that of the intersection of the potential energy surface with a different spin multiplicity, so that the reaction of alkane radical and the oxygen molecule is spontaneous. After the spin multiplicity changes, peroxyl radicals can be generated. In such a case, the reaction between fuel alkyl radical and oxygen is still significantly reversible. In general, the reversibility increases when temperature increases [39]. Jiao et al. detected the generation of ·COOH intermediates during the PE photoconversion under simulated natural environments by conducting in situ FTIR, which was consistent with our calculation.

#### 3.1.3. Reaction of Peroxyl Radical (ROO·) with Alkane or Alkane Radical

The reactions of peroxyl radicals with alkanes or alkane radicals were predicted as follows:

ROO· + RH→ROOH + R· ROO· + R·→2RO·

For the reaction of ROO· with alkanes, the calculated results are shown in Figure S4. The distance between H_41_ and C_25_ in Figure S4C was 2.20 Å. The calculation results suggested that H_41_ transferred from the alkane to the ROO·, generating ROOH and alkane radical. The activation energy of this reaction was 18.15 kcal/mol (equal to 0.0289Ha), which indicates that this reaction can occur at ambient temperature and pressure.

Similarly, the calculated results for the reaction of ROO· with alkane radicals are shown in Figure S5. The distance between the two oxygen atoms, O_20_ and O_21_, in Figure S5C, was 3.83 Å. It is obvious that the O–O bond in the reactant was broken. The calculation results suggested that O_20_ transferred from ROO· to the carbon backbone of the alkane radical, generating an O_20_–C_25_ bond and two alkoxy radicals (RO·). The activation energy of the reaction was 19.45 kcal/mol (equal to 0.0310Ha), which indicates that this reaction can also occur at ambient temperature and pressure. Similar results are reported by Battin et al. They indicated that, under ambient temperature, H atoms are abstracted by alkyl peroxyl radicals from organic molecules, generating alkyl hydroperoxides (ROOH) [40].

The above calculation and analysis indicated that the reaction of ROO· with alkane or alkane free radical under ambient temperature and pressure can occur, generating alkyl hydroperoxides (ROOH) and alkoxy radicals (RO·). However, ROOH molecules are unstable and will be spontaneously converted to lipoperoxyl radicals (ROO·) or under the catalytic conditions of transition metals, such as Fe^2+^. These will continuously react with alkane or alkane radicals [41].

#### 3.1.4. Reaction of Alkoxy Radical (RO·) with Alkane

We studied whether the reaction of oxygen organic free radicals (RO·) with alkanes could take place, as follows:

RO· + RH→ROH + R·

The calculated results are shown in Figure S6. The distance between H_40_ and C_24_ in Figure S6C was 2.40Å. From the calculation results, we found that H_40_ was transferred from the alkane to the RO·, generating ROH and alkane radicals. The activation energy of the reaction was 6.56 kcal/mol, which indicates that this reaction can easily occur under ambient temperature and pressure. 

#### 3.1.5. Reaction of Alkane Radical with Superoxide Anion Radical

The superoxide anion $O_{2}^{-\cdot }$ was another kind of oxygen radical. We calculated whether the reaction of it with alkane radical could occur, as follows:

$$R\text{·}+{\;O}_{2}^{-\cdot }\to {\mathrm{ROO}}^{-}$$

Figure S7 shows the calculated results. The distance between O_21_ and C_4_ in Figure S7C was 1.49Å. It is obvious that the new C_4_–O_21_ bond in the reactant and ROO^−^ were generated. The activation energy of the reaction was 24.94 kcal/mol, which indicates that this reaction can occur slowly under normal temperature and pressure. Therefore, when only the oxygen radical species superoxide anion and hydroxyl radical are present, polyethylene is slowly oxidized.

If H^+^ is present in the reaction environment, the reaction of ROO^−^ with H^+^ occurs and thus ROOH is generated. ROOH is unstable and can spontaneously transform into ROO· or under the catalytic conditions of transition metals, which can continuously react with alkane or alkanes radical. Finally, ROH can be generated.

### 3.2. The Influence of Carbocation on the Strength of PE Carbon Backbone

The above calculations suggest a potential path for PE oxidation. However, the oxidation at a single position cannot cause the C–C bond cleavage because the energy required for the abstraction of a hydrogen atom at different positions is similar. This suggests that the loss of a hydrogen atom can randomly occur. When multiple hydrogen anions are extracted, carbocations are generated. Carbocation, like free radicals, is a very reactive intermediate. The OleT_JE_ (a P450 from the cyp152 family, including bacterial fatty acid hydroxylases) enzyme may produce carbocations in the process of catalyzing fatty acid decarboxylation [42,43]. Therefore, we studied the influence of carbocations on the C–C bond. The carbon backbone with 12 carbon atoms was simulated, in which the hydride ions at different positions were removed. This resulted in a different distribution of carbocations on the carbon chain. The bond orders and flexible force constants of the two weakest C–C bonds, together with the single point energy of the carbon chain containing two carbocations under different distribution conditions, are shown in Table 2. With the amount of carbocations added, the flexible force constants and the bond orders of the two weakest bonds decreased while the bond lengths increased, indicating that the strength of the C–C bond decreased. Thus, this increased the possibility of the carbon backbone scission.

When the calculation results of two carbocations were distributed at different positions on the carbon chain (Table 2), the weakest C–C bond on the carbon chain gradually decreased when the positions of the two carbocations were close to each other (other than two carbocations in the ortho position). Nonetheless, under the aforementioned circumstances, the single point energy of the corresponding molecule gradually increased, indicating that as the density of carbocations increases, it is easier for the cleavage of carbon chain. 

The above calculations show that the existence of carbocations significantly weakens the strength of adjacent C–C bonds in a carbon chain. The likelihood of chain scission markedly increases with the increasing density of carbocations. However, a higher density of carbocations also correlates with a higher overall energy of the entire molecule, which is consequently more difficult to generate. It has been found that, in the process of thermal degradation (150–1000 °C without additional oxygen supply) of PE and other vinyl plastics [44,45], the formation of carbocations was evident in the carbon backbones. As such, this verifies the practical feasibility of our calculation results.

## 4. Conclusions

Using quantum chemical simulations and calculations, we investigated the beneficial effect of various active oxygen radicals (i.e., hydroxyl radicals and superoxide anion radicals) on the oxidation of PE. Hydroxyl radicals were found to easily extract a hydrogen atom from a simulated PE fragment under certain conditions. However, the formation of free alkane radicals is clearly insufficient to reduce the strength of the C–C bond in the polymer backbone, which can result in the chain scission. When exposed to natural light, the alkane radicals can be further oxidized by oxygen, leading to the formation of alcohols and carboxylic acids. Under ambient temperature and pressure, the reaction of alkane radicals with superoxide anions can occur slowly, generating ROO^-^. On the other hand, the presence of carbocations has been found to significantly reduce the strength of several adjacent C–C bonds in a carbon chain, thereby facilitating the chain scission. Although higher densities of carbocations promotes further cleavage of the C–C backbones, the correlated higher energy of entire polymer molecules only allows their presence under extreme conditions. In conclusion, our research provides theoretical knowledge on how and under what conditions the C–C backbone cleavage in PE can occur, allowing for a better understanding of the degradation of plastics with inert C–C backbones.

## Figures and Tables

**Table 1:** Fuzzy bond order of different alkane radicals (the unit is Å).

Bond	Alkane	C4 Position Losing One H Atom	C4 Position Losing Two H atoms	C3 and C4 Position Losing One H atom Respectively	C2 and C4 Position Losing One H atom Respectively
C1–C2	1.17	1.17	1.17	1.16	1.25
C2–C3	1.12	1.11	1.10	1.18	1.19
C3–C4	1.12	1.20	1.27	1.28	1.19
C4–C5	1.12	1.20	1.27	1.19	1.20
C5–C6	1.17	1.16	1.15	1.16	1.16

**Table 2:** The strength of the two weakest bonds in the carbon chain in the presence of carbocations (letters in italic) and the energy of the molecules containing two carbocations.

Number	Schematic Diagram of the Carbocation Distribution (The Positions of Carbocation Are Shown in Bold and Italic)	Flexible Force Constants of the Two Weakest Bonds in the Carbon Chain (mdyn/Å)		Fuzzy Bond Order that Corresponds to the Weakest Two Bonds		Single Point Energy (kcal/mol)
1	C_1_–C_2_–C_3_–C_4_–C_5_–C_6_–C_7_–C_8_–C_9_–C_10_–C_11_–C_12_–R	3.94	4.02	1.12	1.17	-
2	C_1_–C_2_–C_3_–***C_4_***–C_5_–C_6_–C_7_–C_8_–C_9_–C_10_–C_11_–C_12_–R	2.81	1.77	1.11	0.92	-
3	C_1_–C_2_–C_3_–***C_4_***–***C_5_***–C_6_–C_7_–C_8_–C_9_–C_10_–C_11_–C_12_–R	1.54	1.66	0.94	0.93	0
4	C_1_–C_2_–C_3_–***C_4_***–C_5_–***C_6_***–C_7_–C_8_–C_9_–C_10_–C_11_–C_12_–R	1.08	1.13	0.86	0.88	7.61
5	C_1_–C_2_–C_3_–***C_4_***–C_5_–C_6_–***C_7_***–C_8_–C_9_–C_10_–C_11_–C_12_–R	1.25	1.24	0.87	0.87	−5.32
6	C_1_–C_2_–C_3_–***C_4_***–C_5_–C_6_–C_7_–***C_8_***–C_9_–C_10_–C_11_–C_12_–R	1.22	1.33	0.85	0.87	−17.73
7	C_1_–C_2_–C_3_–***C_4_***–C_5_–C_6_–C_7_–C_8_–***C_9_***–C_10_–C_11_–C_12_–R	1.48	1.43	0.89	0.88	−26.54
8	C_1_–C_2_–C_3_–***C_4_***–C_5_–C_6_–C_7_–C_8_–C_9_–***C_10_***–C_11_–C_12_–R	1.60	1.52	0.90	0.89	−33.81
9	C_1_–C_2_–C_3_–***C_4_***–C_5_–C_6_–C_7_–C_8_–C_9_–C_10_–***C_11_***–C_12_–R	1.63	1.59	0.91	0.90	−39.42
10	C_1_–C_2_–C_3_–***C_4_***–C_5_–C_6_–C_7_–C_8_–C_9_–C_10_–C_11_–***C_12_***–R	1.70	1.66	0.91	0.90	−43.66
11	C_1_–C_2_–C_3_–***C_4_***–C_5_–C_6_–***C_7_***–C_8_–C_9_–***C_10_***–C_11_–C_12_–R	0.79	1.00	0.79	0.85	-

## Data Availability

The data presented in this study are available on request from the corresponding author.

## Supplementary Materials

The following are available online at https://www.mdpi.com/article/10.3390/polym13162730/s1, Figure S1: Total energy change as a function of the increasing the distance between O15 and H15 during a relaxed scan based on the structure shown in Figure 1A, Figure S2: IRC analysis potential energy curve, Figure S3: (A) alkane free radical with ortho-carbon atoms each containing an unpaired electron; (B) alkane radical with meta-carbon atoms each containing an unpaired electron, Figure S4: structure of the reactant; (B) The structure of the transition state structure; (C) The structure of the product structure; (D) IRC analysis potential energy curve, Figure S5: (A) The structure of the reactant; (B) The structure of the transition state structure; (C) The structure of the product structure; (D) IRC analysis potential energy curve, Figure S6: (A) The structure of the reactant; (B) The structure of the transition state structure; (C) The structure of the product structure; (D) IRC analysis potential energy curve, Figure S7: (A) The structure of the reactant; (B) The structure of the transition state structure; (C) The structure of the product structure; (D) IRC analysis potential energy curve.
