# Supplementary material for: Quantum Mechanical Investigation of the Oxidative Cleavage of the C–C Backbone Bonds in Polyethylene Model Molecules
Source: Polymers (Basel). 2021 Aug 15;13(16):2730. doi: 10.3390/polym13162730 (PMC8398876; doi:10.3390/polym13162730)
Supplement: Supplementary file 1 [file polymers-13-02730-s001.zip › polymers-1334260-supplementary.pdf]

Quantum mechanical investigation of the oxidative cleavage of the C-C backbone bonds in polyethylene in polyethylene model molecules

Qixuan jiang[1], Zhongyu Li[1], Ziheng Cui[1], Ren Wei[2], Kaili Nie[1], Haijun Xu[1*], Luo Liu[1*]

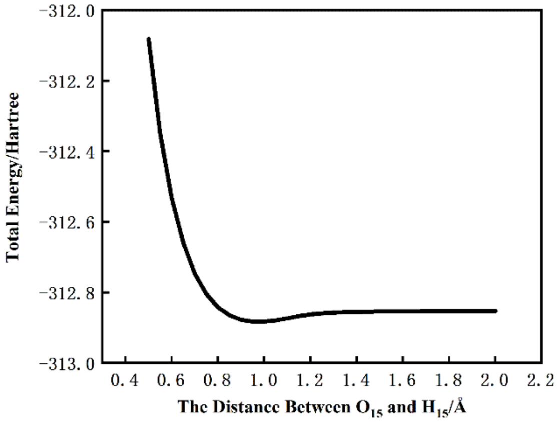

Figure S1 | Total energy change as a function of the increasing the distance between O_{15} and H_{15} during a relaxed scan based on the structure shown in **Figure 1A**. The unit used for the y-axis is Hartree with one Ha equal to 2625.50 kJ/mol.

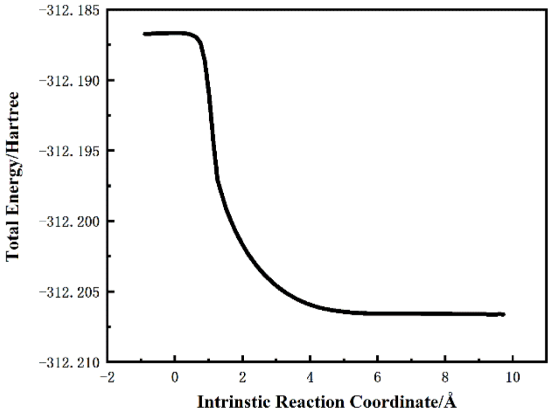

Figure S2 | IRC analysis potential energy curve.

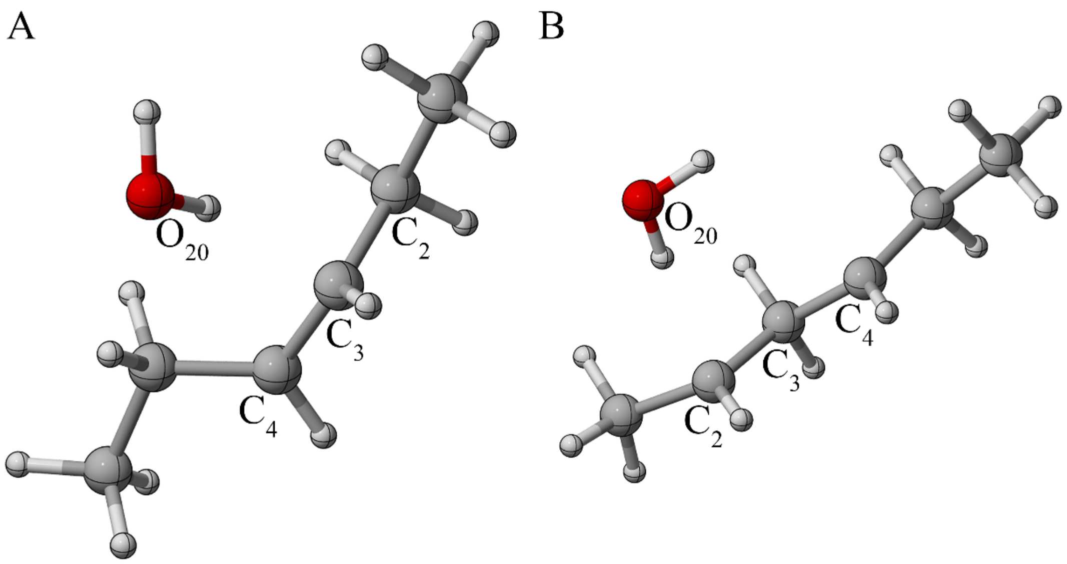

Figure S3 | **(A)** alkane free radical with ortho-carbon atoms each containing an unpaired electron; **(B)** alkane radical with meta-carbon atoms each containing an unpaired electron

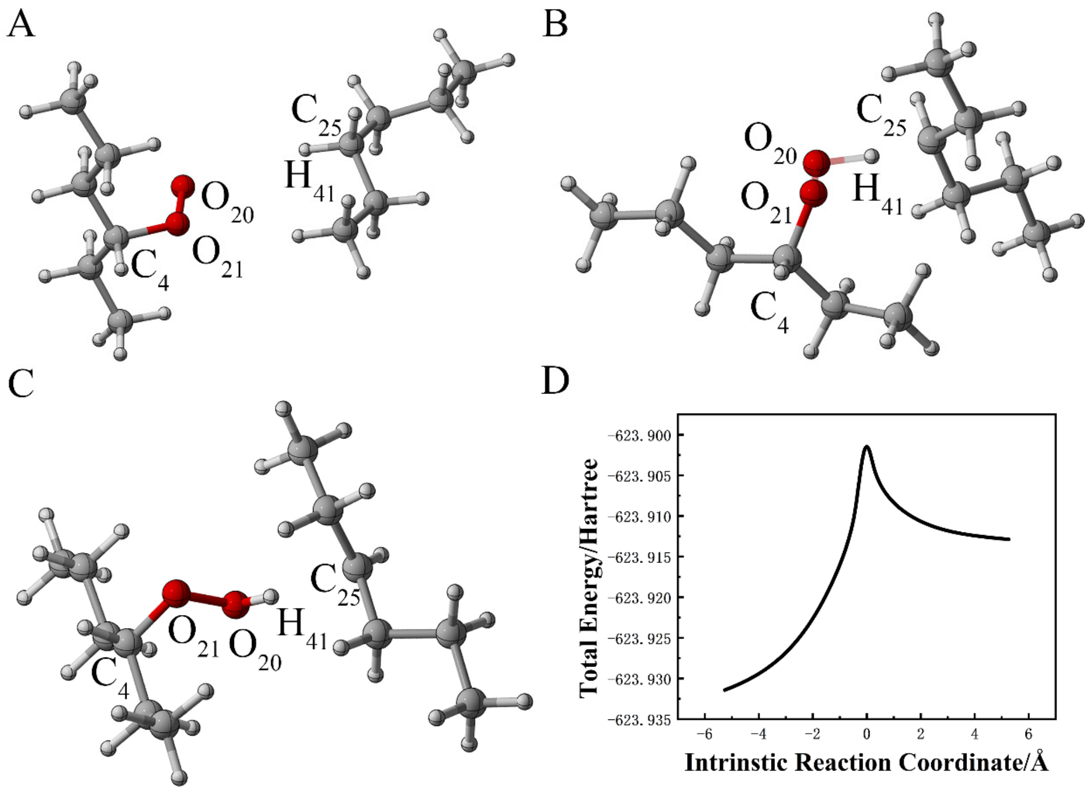

Figure S4 | **(A)** The structure of the reactant; **(B)** The structure of the transition state structure; **(C)** The structure of the product structure; **(D)** IRC analysis potential energy curve.

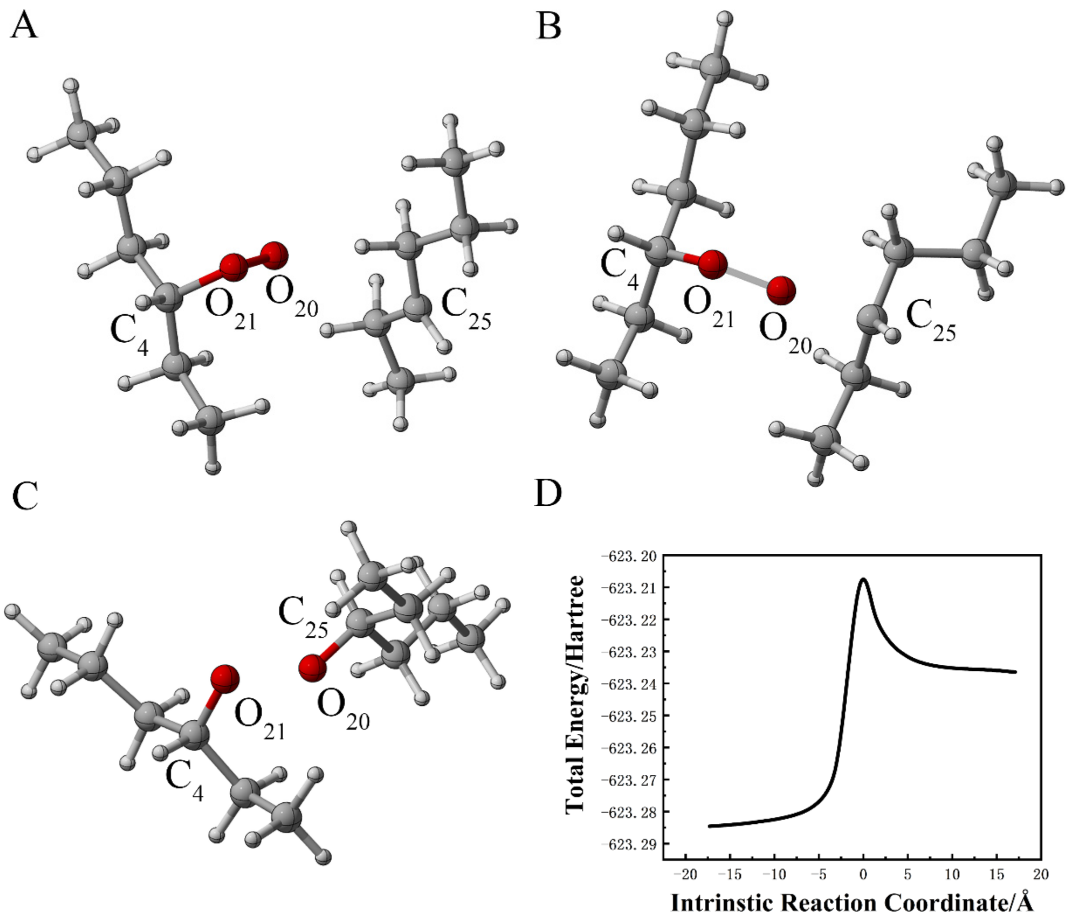

Figure S5 | **(A)** The structure of the reactant; **(B)** The structure of the transition state structure; **(C)** The structure of the product structure; **(D)** IRC analysis potential energy curve.

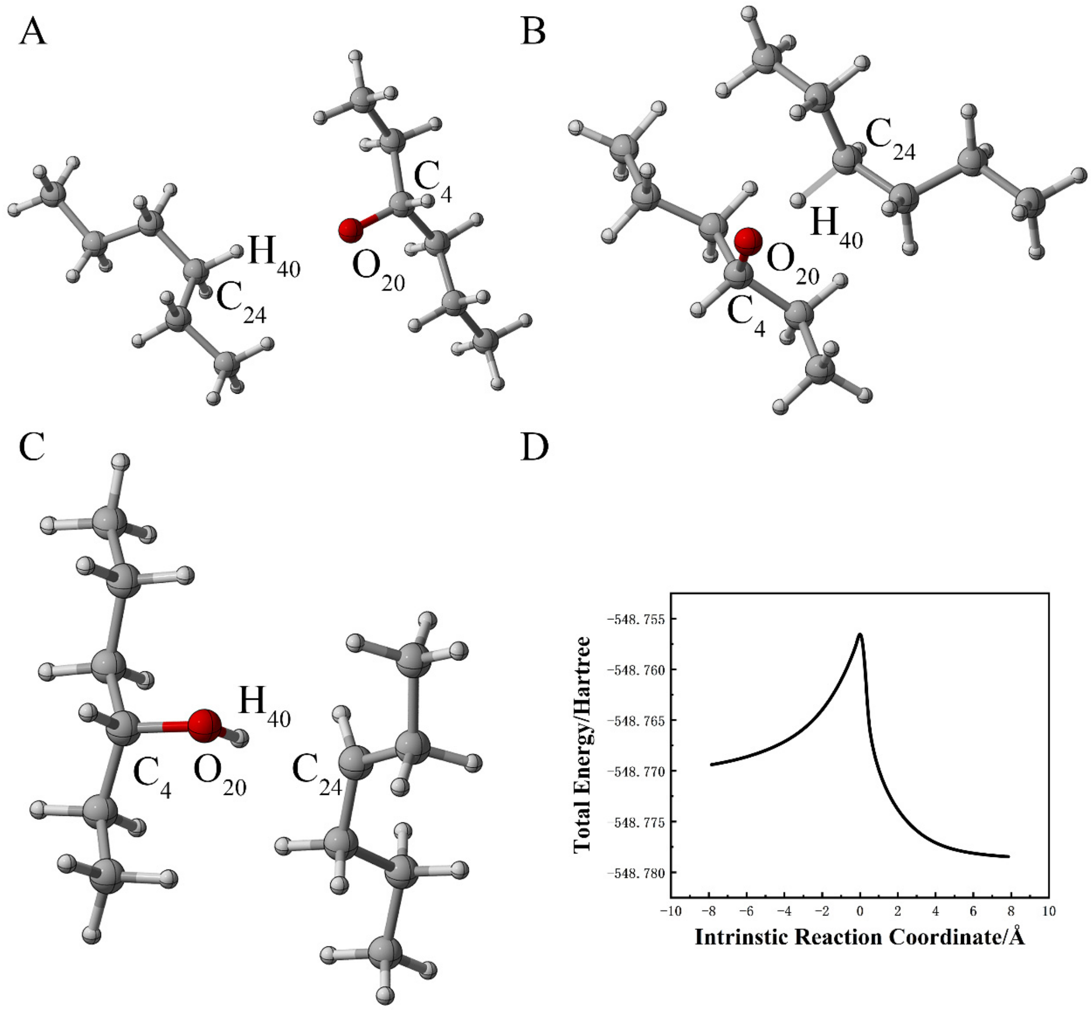

Figure S6 | (A) The structure of the reactant; **(B)** The structure of the transition state structure; **(C)** The structure of the product structure; **(D)** IRC analysis potential energy curve.

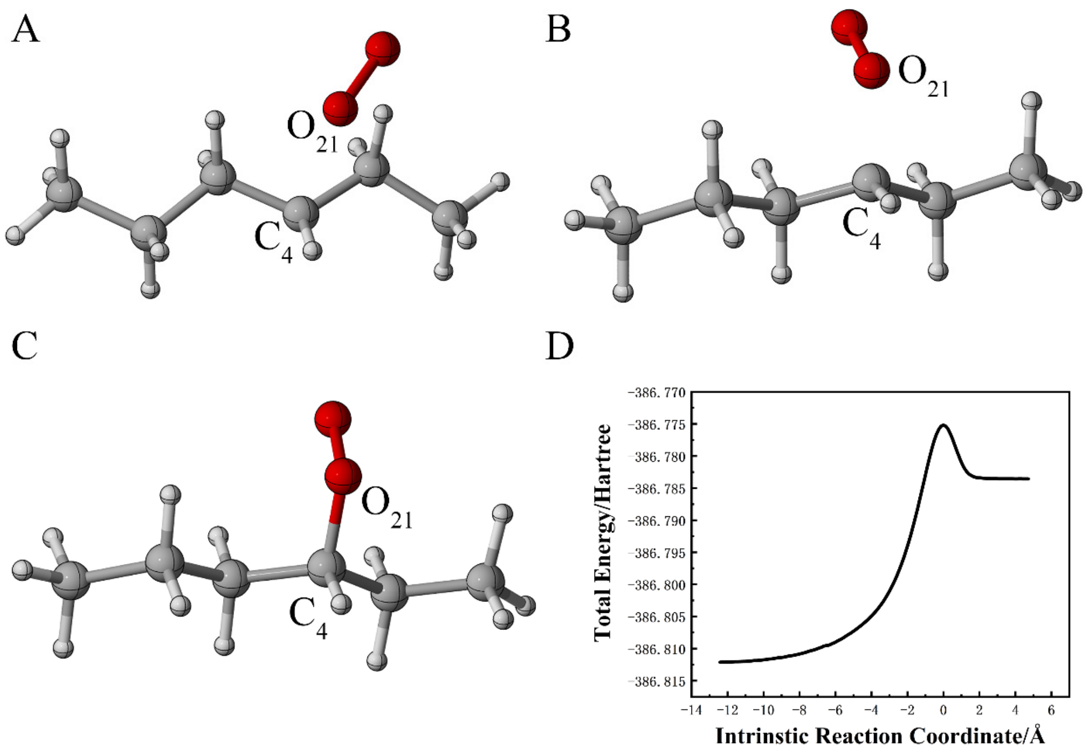

Figure S7 | (A) The structure of the reactant; **(B)** The structure of the transition state structure; **(C)** The structure of the product structure; **(D)** IRC analysis potential energy curve.
